# Usability, adherence and efficacy of coach-augmented dHealth cognitive behavioural therapy for insomnia in South African sexual assault survivors: a pilot study

**DOI:** 10.3389/fpsyt.2026.1651928

**Published:** 2026-09-08

**Authors:** Gosia Lipinska, Julia Boland, Sage E. Seef, James A. A. Rink

**Affiliations:** Applied Cognitive Science and Experimental Neuropsychology Team (ACSENT) & UCT Sleep Sciences, Department of Psychology, University of Cape Town, Cape Town, South Africa

**Keywords:** CBT-i, coach support, dHealth, posttraumatic stress disorder, sleep, sleep hygiene, sleep rescheduling, stimulus control

## Abstract

**Introduction:**

Treating sleep difficulties - a core symptom of posttraumatic stress disorder (PTSD) - can improve both sleep and broader psychiatric outcomes. Digital health (dHealth) applications offer a scalable way to deliver cognitive behavioural therapy for insomnia (CBT-i), particularly for trauma survivors facing barriers to care. However, adherence to these interventions is often poor and may be improved through human support, especially for more demanding components such as sleep rescheduling. This study evaluated the usability, adherence, and preliminary efficacy of a dHealth application delivering sleep rescheduling both with and without coach support, alongside self-guided stimulus control and sleep hygiene components.

**Methods:**

Female sexual assault survivors were assigned to one of two groups comparing the sleep rescheduling component of the dHealth application: either coach-assisted (n = 27) or self-guided (n = 27). All participants received stimulus control and sleep hygiene information embedded in the application, in the form of a newsletter, including a third comparator group (n = 25), which received this information only. Usability and negative effects were self-reported. Adherence was assessed via dropout rates, application engagement, newsletter openings, and compliance with sleep prescriptions. Efficacy was measured by changes in insomnia, depressive, and PTSD symptoms.

**Results:**

The self-guided group reported lower satisfaction and more negative effects than controls. Compared to the self-guided group, coach-assisted participants showed better adherence to sleep prescriptions. Across groups, insomnia and depressive symptoms decreased significantly. Preliminary evaluation of efficacy showed that PTSD symptoms improved substantially in the coach-assisted and comparator groups, while improvements in the self-guided group plateaued after initial gains.

**Discussion:**

Unguided application use was associated with poorer usability, greater negative effects, lower adherence, and some potential weaker outcomes. Preliminary findings suggest that sleep rescheduling may benefit from human support to be effective. Where coaching is unavailable, simple stimulus control and sleep hygiene education may be preferable.

## Introduction

In low- and middle-income countries (LMICs) such as South Africa, gender-based violence is highly prevalent. A cross-national survey of 57 countries reported that South Africa had the highest rates of sexual assault (1, 2). Female tertiary students are particularly vulnerable, with markedly higher rates of gender-based violence (74.20%) and sexual assault (26.87%) compared to both male peers and other age groups (3, 4). Sexual assault is associated with a wide range of short- and long-term health consequences (5, 6), including sexually transmitted infections, unintended pregnancies, physical injuries, and mental health problems (7). Among these, posttraumatic stress disorder (PTSD) is especially debilitating; in South Africa, survivors of sexual assault are up to five times more likely to develop PTSD than women exposed to other forms of trauma (8).

PTSD is associated with a range of physical, cognitive, and affective symptoms, including sleep disturbance that affects daytime functioning. Recent studies emerging from our laboratory find that > 90% of participants who have experienced sexual assault report significant sleep disruption at > 6 months post-trauma (9).

Moreover, sleep therapy is emerging as a novel and beneficial strategy for the secondary and tertiary prevention of psychiatric disorders, including PTSD (10, 11). This is because emergent literature demonstrates that disordered sleep is not only a consequence of trauma exposure but may also drive pathophysiological changes associated with the development of PTSD (12, 13). For example, several studies demonstrate that those who experience poor sleep quality in the aftermath of a traumatic event are significantly more likely to present with PTSD symptoms at 6 weeks (14) or even 1-year follow-up (15). There are also mechanistic relationships between sleep disruption and other symptoms of PTSD. Specifically, the degree of rapid eye movement (REM) sleep fragmentation predicts sleep-dependent memory consolidation of neutral information in individuals who endorse PTSD symptomology but not in control participants (16, 17). Because REM sleep, in particular, is critical for fear extinction (18, 19), disruption at that stage of sleep in PTSD-symptomatic individuals is also likely to contribute to disturbances in fear regulation, the core symptom of PTSD (19). During REM sleep, PTSD-symptomatic individuals show hypermetabolism in networks associated with arousal regulation and fear response, suggesting that the emotion regulatory benefits that REM sleep usually confers (12) are interrupted in this clinical population. These findings emphasise that sleep difficulties in PTSD are not isolated or secondary symptoms, but that they are meaningfully related to core features of the disorder.

Supporting the hypothesis that sleep difficulties are predictably related to other symptoms of PTSD, empirical studies have indicated that several therapies designed to improve sleep in PTSD also alleviate some other symptoms (20, 21). These therapies include behavioural treatments such as image rehearsal therapy, and cognitive behavioural therapy for insomnia (CBT-i), which includes components such as sleep rescheduling and stimulus control (21–23). However, these therapies are all resource- and time-intensive because they require in-person consultation with a trained medical professional.

Furthermore, in many LMIC settings like South Africa, sexual assault survivors have limited access to mental health services. Significant barriers to access include cost of services, stigma and discrimination associated with seeking mental health care, lack of knowledge about mental illness, sociocultural beliefs, and structural barriers such as transportation costs (24, 25). Female tertiary students are likely to be affected by all of these factors, and there is evidence that students have extremely low attendance at outpatient clinics in South Africa (24, 26). Where services are more accessible (e.g. university in-house care), these services are overburdened and characterised by long waiting lists, limiting timely access. This confluence of limited availability and barriers to accessibility results in many women being isolated with posttraumatic symptoms with little prospect of relief.

Research suggests that some internet-based and digital health (dHealth) adaptations of behavioural sleep therapies are as effective as in-person treatment (27). In the Global North, dHealth applications are increasingly popular due to their accessibility, cost-effectiveness, time efficiency, and scalability. In South Africa, 96.1% of households own at least one smartphone, and smartphone penetration is among the highest globally (28). Furthermore, 75.6% of internet use occurs via smartphones (28), making them the primary means of online access for many individuals. As a result, dHealth applications represent a practical and scalable approach for delivering widely accessible treatment.

A number of existing dHealth applications are designed to treat sleep difficulties. Applications such as CBT-i Coach, Sleepio, and Sleep Sensei have substantial evidence supporting their efficacy (29–32). For example, a randomised controlled pilot of CBT-i Coach showed that the application significantly augmented in-person CBT-i treatment for participants with moderate-severe insomnia (30). CBT-i Coach was effective for reducing insomnia symptoms in a review examining the efficacy of 10 other mobile tools (31). Furthermore, Sleepio was proven to be efficacious in a randomised control trial, with a significant decrease in insomnia severity after 10 weeks (32). Similarly, Chan and colleagues (29) found that Sleep Sensi was efficacious in treating insomnia, with improvements largely sustained at 4-week follow-up.

There are, however, some disadvantages related to using these applications to improve sleep quality. Primary amongst these is the high attrition rate (approximately 50%) associated with application use (33). The rates for such applications are likely to be even higher in LMIC countries where (a) there is no strong ‘self-help’ culture (34), and (b) multiple competing demands on time and resources mean self-help technologies are likely to be a low priority for most individuals. In fact, there is strong evidence that applications like these would be strengthened by incorporating human interaction into their design as a potential means to overcome high attrition rates (35, 36). These attrition rates may also be overcome when the digital intervention focuses on the components of CBT-i that are most acceptable and effective for users. Specifically, several studies have shown that sleep rescheduling, a technique used to modify the available opportunity for sleep, is difficult to implement and may require the assistance of a therapist or coach (37). Other components are considered easier to implement, including stimulus control, which is designed to strengthen the conditioned association between the bed and sleep, and sleep hygiene, which involves behavioural and environmental modifications that promote sleep. Both are typically included in CBT-i based dHealth applications.

Our pilot study therefore evaluated a dHealth mobile intervention for sleep disturbances in PTSD, focusing on evaluating coach-assisted sleep rescheduling for user experience, adherence, and preliminary efficacy, in the context of standard stimulus control and sleep hygiene information.

We hypothesised that sexual assault survivors with sleep difficulties and posttraumatic symptoms, who use a coach-assisted version of a dHealth sleep rescheduling application, compared to those who use a self-administered version, will show:

- greater usability and fewer negative side effects;
- higher adherence to the application (≥ 50%); and
- greater improvements in posttraumatic symptoms, sleep quality, and depressive symptoms.

## Methods

### Participants

Women aged 18–40 were recruited from host institutions via email advertisements distributed through institution-wide networks. All participants reported experiencing sexual assault more than three months prior, alongside posttraumatic symptoms and sleep disturbance. Exclusion criteria included: (a) sexual assault or other DSM-5 Criterion A trauma (38) within the past three months (including ongoing trauma, such as domestic violence or cohabitation with the perpetrator); (b) moderate-to-high suicide risk; (c) neurological conditions affecting sleep (e.g., epilepsy); (d) use of psychoactive medication for less than three months; (e) use of hypnotics; (f) pregnancy or lactation; (g) night-shift work; and (h) a history of psychotic or bipolar disorders.

Eligible participants were pseudo-randomly assigned to one of three groups. All groups received standard stimulus control and sleep hygiene information embedded in the information section of the application, in the form of a newsletter that standardised the exposure to this component for all participants. One of the three groups received this intervention only (Newsletter), while the other two additionally engaged with the sleep rescheduling component, either completed independently (App-only) or with coach support (Coach-assisted).

All participants provided informed consent in two stages: first for screening and then for participation in the intervention. The study was approved by the institutional Psychology Ethics Committee and conducted in accordance with the Declaration of Helsinki (39).

A total of 1635 potential participants completed the screening. Of these, 1555 self-identified as female. Smartphone access was unavailable to 9 participants and 94 lacked internet access. There were 138 potential participants who were excluded as they had experienced a trauma within the last 3 months, and a further 437 participants were excluded for having a diagnosis of a neurological condition, taking psychoactive medication, taking hypnotics, being pregnant or lactating, doing shift work, or for having a history of psychotic disorders. Of the 877 eligible participants contacted, 118 expressed interest. The researchers assessed 59 individuals for risk of suicide, of whom 8 were deemed moderate-high risk and were referred to a specialist. Some participants stopped responding for various reasons before recruitment, resulting in a final sample size *N* = 79.

Regarding power analysis, previous randomised controlled trials using dHealth interventions reported moderate between-group effects for sleep disturbance (*d* = .40–.50) (40, 41) and PTSD symptoms (*d* = .41–.54) (42, 43). Therefore, using an average effect size of *d* = .46, power of .80 and α = .05, the required sample size was estimated to be *N* = 36 participants (44) for between-group and within-group comparisons. However, given the high rate of attrition associated with dHealth interventions, the required sample size was adjusted to *N* = 72.

### Materials

#### Screening and efficacy measures

##### Life Events Checklist for DSM-5 (LEC-5)

The LEC-5 (45) was used to exclude individuals who had not experienced sexual assault. This 17-item self-report scale assesses lifetime exposure to potentially traumatic events known to be antecedents of PTSD symptoms. Respondents indicate their exposure using a 6-point nominal scale: “happened to me” (1), “witnessed it” (2), “learned about it” (3), “part of my job” (4), “not sure” (5), and “doesn’t apply” (6). Respondents may endorse multiple levels of exposure for a given traumatic event. Individuals who did not endorse a score of 1 for items 8 or 9—concerning sexual assault or other unwanted or uncomfortable sexual experiences—were excluded from the sample.

Previous editions of the LEC-5 have well-established psychometric properties (46) and only minor revisions have been made since earlier editions, suggesting that the LEC-5 performs similarly.

##### Short PTSD Rating Interview (SPRINT)

The SPRINT (47) was used to select participants into the study and characterise changes in PTSD symptoms in all groups during the experimental phase. This self-report questionnaire comprises 8 items that measure core PTSD symptoms (intrusion, avoidance, numbing, and hyperarousal), somatic problems, stress vulnerability, and social and work-related functional impairment. Items are scored on a 5-point scale, from ‘not at all’ (0) to ‘very much’ (4), and scores are summed to a maximum total of 32. For general population samples, a threshold score in the range 14–17 can be used to distinguish between respondents with and without PTSD and potential participants who scored < 14 were excluded.

The SPRINT has demonstrated convergent validity with the Clinician-Administered PTSD Scale for DSM-IV (CAPS; *r*_s_[18] = .781, *p* <.001) (48), and satisfactory internal consistency in student samples (α = .84) (49). In the current sample, the SPRINT’s internal consistency was unstable at screening (α = .49) but acceptable at 3-days (α = .77), 7-days (α = .82), and 14-days (α = .89).

##### Insomnia Severity Index (ISI)

The ISI (50) was used both to screen participants for inclusion in the study (individuals with ISI scores < 8 were excluded at screening) and to assess sleep disturbance across all groups during the experimental phase. This brief 7-item questionnaire measures the severity of difficulties with sleep onset, sleep maintenance, and early-morning awakening over the previous two weeks. Items are rated on a 5-point scale ranging from ‘no problem/does not apply’ (0) to ‘very severe problem/highly applicable’ (4). Scores are summed and interpreted using the following thresholds: no clinically significant insomnia: 0–7, subthreshold insomnia: 8–14, moderately severe clinical insomnia: 15–21, and severe clinical insomnia: 22–28.

The ISI has demonstrated strong psychometric properties in student samples, with an internal consistency of α = .90 (51). In the current sample, internal consistency was lower at screening (α = .51) but improved at 3 days (α = .73), 7 days (α = .75), and 14 days (α = .89).

##### Patient Health Questionnaire-9 (PHQ-9)

The 9-item PHQ-9 (52) was used to characterise depressive symptoms at baseline and during the study. Respondents indicated the frequency with which they had experienced 9 core symptoms of depression over the past two weeks. Items are scored on a 4-point scale: ‘not at all’ (0), ‘several days’ (1), ‘more than half the days’ (2), and ‘nearly every day’ (3). Depression severity is interpreted according to the following thresholds: minimal depression: 1-4, mild depression: 5-9, moderate depression: 10-14, moderately severe depression: 15-19, and severe depression: 20-27.

The PHQ-9 has demonstrated convergent validity with the Depression Anxiety and Stress Scale-21 (DASS-21; *r_s_*[582] = .69 for depression; *r_s_*[582] = .60 for anxiety; *r_s_*[582] = .62 for stress), and satisfactory internal consistency in student samples (α = .83) (53). In the current sample, the PHQ-9’s internal consistency was acceptable at all points of measurement: screening (α = .81), 3-days (α = .84), 7-days (α = .89), and 14-days (α = .92).

##### Columbia-Suicide Severity Rating Index (C-SSRI)

Based on research showing that the PHQ-9 item 9 does not adequately assess current suicidality (54), we additionally screened participants who endorsed item 9 (score > 0) with the C-SSRI primary care version (55) to determine suicide risk. All members of the research team were trained to administer this measure following the guidance of the developers of the scale (see http://c-ssrs.trainingcampus.net). This questionnaire asks 6 yes/no questions evaluating suicidal ideation, intent, and behaviour. Participants who answered *yes* to C-SSRI questions about specific intent or suicidal behaviour in the last 3 months were excluded from participation and referred to appropriate sources. Participants who declined to complete the C-SSRI were also excluded.

According to the authors, the C-SSRI is sensitive to change over time and has high sensitivity and specificity for suicidal behaviour classifications in comparison to other behaviour scales and to independent suicide evaluation by a panel of clinicians.

##### General Screening Questionnaire

A general screening questionnaire excluded respondents based on recent traumatic experiences (< 3 months prior to screening), sleep-influencing neurological conditions, history of psychosis or bipolar depression, night-time shift work, pregnancy, age (< 18, > 40), and sex (male).

#### Intervention conditions

##### CBT-i Coach

The CBT-i Coach mobile application was selected as the dHealth intervention. It was developed as a collaborative effort between the US Veterans Affairs’ National Centre for PTSD, the Stanford School of Medicine, and the US Department of Defence’s National Centre for Telehealth and Technology (56, 57). The study intervention used only the core functions of the application. Participants completed daily sleep diary entries and used the application to schedule sleep-related behaviours, including prescribed bed and wake times, cut-off times for caffeine intake, designated worry periods, and wind-down routines. After at least five diary entries, the application generates a sleep prescription, which allows participants to apply sleep rescheduling, to help entrain and consolidate their sleep to their preferred schedule. Participants were instructed not to use other application functions (e.g. guided meditations and in-depth sleep-related information) to eliminate possible confounds.

##### Coach-assistance

Participants assigned to the Coach-Assisted arm of the study used the CBT-i Coach application as described above but also benefitted from an online coach who helped orient participants to the application and guide them with regards to its use. The coaches placed specific emphasis on supporting participants in the application of the sleep prescription.

##### Email Newsletter

A total of 14 daily email newsletters were delivered to all participants during the intervention period. Each newsletter contained information drawn from the ancillary sections of the CBT-i Coach application, which focus on stimulus control and sleep hygiene. Each email was short, instructive, and sent via the Mailchimp online email service platform (58).

#### Intervention measures

##### Negative Effects Questionnaire (NEQ)

An adapted version of the 32-item NEQ (59) measured, both related and unrelated to the intervention adverse events (AEs) experienced during the intervention. All questions in the original NEQ that refer to a ‘therapist’ or ‘treatment’ were changed to refer instead to ‘the application’ or ‘the newsletter’. Participants rated the extent to which they experienced AEs on a 5-point scale from: ‘not at all’ (0) to ‘extremely’ (4). Respondents were asked to differentiate between negative effects they would attribute to the intervention and those likely caused by other circumstances.

In the current sample, the NEQ’s internal consistency was acceptable both overall (α = .90) and for negative effects attributed to the intervention (α = .89).

##### System Usability Scale (SUS)

A modified version of the 10-item SUS (60) tracked the usability of the dHealth application. The SUS can assess various products and services, including hardware, software, and web and mobile applications. It measures self-reported user experience, including ease of use, learning curve, confidence in the system, and consistency and integration of system functions, which are reported using a 5-point scale from ‘strongly disagree’ (0) to ‘strongly agree’ (4). A SUS score is calculated by summing the scores of odd items and the reverse-scores of even items, before multiplying by 2.5, providing a maximum SUS score of 100.

Mean SUS scores are normalised as percentile ranks and compared with benchmark ranks established for comparable systems (61). For mobile applications, the mean SUS score for the 10 most popular applications on Apple and Android platforms was *M* = 81.00 (SD = 16.40) (62, 63).

In terms of reliability, ten separate studies conducted between 2008 and 2018 reported satisfactory internal consistency for the SUS (α = .83–.97) (60). In the current sample, the SUS’ internal consistency was acceptable (α = .78).

##### Telehealth Usability Questionnaire (TUQ)

A modified version of the TUQ (64) tracked usability factors associated with the dHealth intervention. The TUQ is a composite of several existing telehealth and information technology questionnaires (65–67), intended to measure the usability of videoconferencing systems, computer-based systems, and mobile telehealth systems using 5 subscales: usefulness, ease of use, effectiveness, reliability, and satisfaction. The 21 items are measured on a 7-point scale from ‘disagree’ (1) to ‘agree’ (7). Although no formal scoring procedures have been published, TUQ results are reported in terms of mean scores for each item, subscale, and total score, where higher mean scores indicated better system usability (68).

The TUQ subscales have demonstrated good reliability and validity, with α = .81–.93 and unidimensional factor loadings that account for large proportions of variance (.66–.82) (64). In the current sample, the TUQ’s internal consistency was acceptable (α = .90).

### Procedure

#### Screening, baseline and randomisation

After completing the screening survey, eligible participants were randomly assigned to the Coach-assisted, App-only or the Newsletter group using a random-number-generator (69). When participants endorsed suicidality on the PHQ-9, a researcher completed the C-SSRI with them via teleconferencing. Participant screening responses on the ISI, PHQ-9 and SPRINT were used as baseline scores for eligible participants.

#### Intervention

The intervention stage of the study proceeded in the following order: (a) informed consent, orientation, and exposure to intervention condition (*t*_0_), (b) assessments 3 days, 7 days, and 14 days after exposure, and (c) debriefing. All participants received an introductory email outlining their intervention. Participants in the App-only and Newsletter groups completed their respective consent forms individually and then viewed a series of written tutorials that oriented them with their assigned conditions. Participants in the Coach-assisted group received an online individual virtual meeting with a researcher who guided them through their consent form and orientation tutorials, which included explanation of the active component of sleep rescheduling. This explanation focused on providing information about how to apply the sleep prescription and to inform the participant about the way in which this component of CBT-i functioned. The Newsletter group orientation included only information on the newsletter subscription procedure. All participants were reminded that the newsletters would contain educational information intended to assist with sleep difficulties. All three orientations included a direct download/subscription link to their respective condition at the end of the tutorials, to ensure that participants were exposed to their assigned condition with an approximately equivalent level of information.

Participants in all three groups were requested not to conduct additional research or download additional resources to assist with sleep.

##### Coach-assistance during the intervention period

Participants in the Coach-assisted group received support from one of two trained sleep coaches. Sleep coaches were postgraduate students, with experience working on clinical projects. They were trained by G.L., a registered Neuropsychologist with extensive experience in sleep medicine. Training involved role-play scenarios and feedback, until the coaches were confident to provide support to participants. Throughout the study, the research team held weekly case conferences to review coach support on a case-by-case basis, allowing adjustments based on supervision feedback. Support consisted of daily WhatsApp check-in messages sent within one hour of waking to facilitate use of the CBT-i Coach application. Participants were asked about any difficulties with the application and their ability to follow the prescribed sleep schedule. When implementation challenges arose, the sleep coach provided guidance on adapting sleep environments and routines to improve adherence. The coach did not discuss trauma-related or other personal psychological content with participants; where such support was required, participants were referred to appropriate external resources.

##### Assessments and Debriefing

There were 3 assessments after exposure, measured at (a) *t*_0_ + 3 days, (b) *t*_0_ + 7 days, and (c) *t*_0_ + 14 days, administered as web surveys. The questionnaires in each survey included the ISI, SPRINT, and the PHQ-9. The PHQ-9 was adapted so that it corresponded to the period under assessment. For example, participants would be asked to rate their depressive symptoms in the last 3, 4 and 7 days, rather than the last two weeks.

For the final assessment at *t*_0_ + 14 days, the SUS, TUQ, and NEQ were also included in the web surveys. Participants in the Coach-assisted and App-only groups were asked to export their user data from the CBT-i Coach application on each of the 3 assessment days.

Additionally, in each assessment, every participant was asked whether they had conducted additional research or downloaded resources to assist with sleep (other than CBT-i Coach for the intervention groups), and were reminded not to expose themselves to these additional resources. Participants in the Coach-assisted and App-only groups were also reminded not use any additional components of the CBT-i Coach application.

The final survey ended with a debriefing document and provided participants in the Newsletter group the opportunity to download the CBT-i Coach application.

### Statistical analysis

#### Data imputation and transformation

In terms of negative effects, system usability, and telehealth usability, 7 data-points from 7 participants were missing at the 14-day time point. In terms of insomnia, depression, and posttraumatic stress, no data were missing at baseline. However, a total of 24 data-points from 14 participants were missing for clinical variables measured after baseline. These missing data were imputed using predictive mean matching. This method deviates from a linear imputation in that predicted values are compared to existing values. Rather than all lie on a prediction path, noise is added by pulling predictions towards other data values, based on shared characteristics in non-missing variables. This retains the data’s distribution, and reduces risk of introducing linear dependencies in the data. The presence of non-normal distributions recommended this method (70). Variable means changed by between –0.2 and 0.2, indicating minimal biasing. The distributions were approximately normal, post imputation.

Usability and adherence were the primary study outcome variables and intervention efficacy were analysed as secondary pilot data. Analyses were conducted in SPSS v30 (71) and Rstudio (72), and alpha was set to 0.05. Where analyses included multiple comparisons, *p*-values were adjusted using the relevant statistical adjustment.

#### Sociodemographic differences at baseline

All groups were tested for significant differences in sociodemographic variables (age, household income category, and number of persons per household). For continuous variables, one-way ANOVA was used, and for categorical variables, chi-squared tests of contingency were used.

#### Usability

Usability outcomes assessed at 14 days were analysed for between-group differences. These included reported negative effects during the study, attribution of such effects to the intervention or other causes, and overall system usability, along with subscale scores from the TUQ. Statistical comparisons of all metrics between groups excluded items that were unique to a subset of groups; e.g. there were some usability items specific to coaching that did not apply to the other groups. One-way ANOVA was used for all analyses.

#### Adherence

The user data exported by participants in the intervention groups were used to provide descriptive statistics for the number of sleep diary entries, the number of completed study days, and adherence to the sleep prescription.

Engagement with the newsletter was measured by registering the newsletter as (a) opened and, (b) recording at least a one click (58). Between-group analyses were conducted using one-way ANOVA when all interventions were considered and t-tests when the App-only group was compared to the Coach-assisted group.

#### Efficacy

Linear Mixed effect Models were used to test for significant differences in symptoms of posttraumatic stress, insomnia, and depression across 4 time points (baseline, day 3, day 7, day 14). These included time point, group and their interaction. A random intercept for participant was used to control for individual differences in the repeated measures. Significant main effects were followed up with a Tukey-Kramer Honestly Significant Differences (HSD) post-hoc test for group comparisons and a Tukey HSD post-hoc test for time comparisons. Interaction effects were followed up with simple effects analyses, with respective Tukey-Kramer HSD and Tukey HSD post-hoc tests.

## Results

A total of 79 participants were enrolled into the study (App-only: *n* = 27, Coach-assisted: *n* = 27; Newsletter: *n* = 25). Participants were, on average, 21.11 years old (*SD* = 3.51 years) and came from households with *M* = 4.87 (*SD* = 1.87) individuals living in each household. Participants came from homes with a wide spread of income levels (ZAR/month): > R58,918 (high: 40.51%, *n* = 32), R16,876–R58,917 (middle; 31.65%, *n* = 25), and < R16,875 (low; 27.85%, *n* = 22).

In line with eligibility criteria, all participants endorsed at least sub-clinical levels of insomnia and PTSD. Both conditions approached clinical levels of severity in the sample (for insomnia, *M* = 14.18, *SD* = 3.67; for PTSD, *M* = 20.15, *SD* = 4.16), and rates of clinical endorsement were high (for insomnia, 46.84%, *n* = 37; for PTSD, 94.94%, *n* = 75). Depression also approached a clinical level of severity in the sample, *M* = 14.61 (*SD* = 5.41), and the rate of endorsement was the same as that for insomnia, 46.84% (*n* = 37). The most commonly identified timeframe for worst lifetime traumatic experience was in the past 1–5 years (44.3%, *n* = 35), and the three most common experiences were *Other unwanted or uncomfortable sexual experience* (97.47%, *n* = 77), *Transportation accident* (for example, car accident, boat accident, train wreck, plane crash; 84.81%, *n* = 67), and *Sexual assault* (rape, attempted rape, made to perform any type of sexual act through force or threat of harm; 83.54%, *n* = 66).

### Sociodemographic differences at baseline

Analyses indicated no significant group-differences at baseline for (a) age, *F*(2,72) = 3.21, *p* = .470, η^2p^ = .021; (b) household income category, χ^2^(4) = 9.99, *p* = .061, *V* = .238; or (c) the number of persons per household, *F*(2,47.62) = 1.82, *p* = .173, η^2p^ = .033.

### Usability

**Table 1** shows group effects for all usability variables.

**Table 1:** Usability ratings of participants completing an App-only, Coach-assisted or Newsletter intervention.

Variable	App-only	Coach-assisted	Newsletter				
	*M (SD)*	*M (SD)*	*M (SD)*	*F*	*df*	*p*	η^2p^
Negative effects ^a^							
Overall ^b^	43.89 (15.31)	38.43 (12.89)	36.28 (12.83)	1.96	2,75	.297	.050
Attributed to intervention ^c^	8.00 (7.90)	4.32 (5.13)	2.27 (2.81)	6.76	2,76	.004	.151
System usability ^b^	78.24 (12.88)	78.10 (10.21)	78.83 (12.76)	0.03	2,75	.974	.001
Telehealth usability ^a^							
Overall ^b^	4.93 (0.74)	5.24 (0.83)	5.22 (0.91)	1.13	2,75	>.999	.029
Usefulness ^c^	3.19 (1.09)	3.56 (1.43)	3.97 (1.41)	2.29	2,76	.648	.057
Ease-of-Use ^d^	5.90 (0.80)	5.97 (0.78)	6.03 (0.73)	0.19	2,74	>.999	.005
Quality ^b^	5.41 (0.95)	5.54 (0.95)	5.74 (0.91)	0.82	2,75	>.999	.021
Reliability ^b^	5.15 (1.01)	5.74 (1.08)	4.44 (1.08)	9.56	2,75	.001	.203
Satisfaction ^c^	4.76 (1.23)	5.47 (1.05)	5.75 (1.16)	5.20	2,76	.046	.120

Means and standard deviations are based on raw scores. For Negative effects attributed to the intervention, statistics are based on offset-logarithmic-transformed scores; *log*_10_(*x* + 1).

^a^ *p*-values adjusted for multiple comparisons; ^b^ *n* = 78; ^c^ *n* = 79; ^d^ *n* = 77.

#### Negative effects

Overall negative effects. ANOVA yielded a non-significant result for differences in overall NEQ scores between groups, as shown in **Table 1**.

Negative effects attributed to the intervention. In this analysis, overall NEQ responses were filtered by attributions to the intervention in each group. This means that only NEQ scores participants attributed to either the newsletter or application were included.

The ANOVA detected a significant, large effect for group differences in NEQ scores, as shown in **Table 1**. A Tukey-Kramer HSD post-hoc test revealed that NEQ scores in the App-only group (*M* = 0.78, *SD* = 0.43) were significantly higher compared to the Newsletter group (*M* = 0.36, *SD* = 0.37, *p* = .001, *d* = 1.02), but not significantly higher compared to the Coach-assisted group (*M* = 0.55, *SD* = 0.42, *p* = .108, *d* = 0.56).

#### System usability

The ANOVA yielded a non-significant result for differences in SUS scores between groups, as shown in **Table 1**. Notably, mean SUS scores in **Table 1** were close to that reported for the 10 most popular applications on Apple and Android platforms (*M* = 81, *SD* = 16.40).

#### Telehealth usability

Overall telehealth usability. The ANOVA yielded a non-significant result for differences in overall TUQ scores between groups, as shown in **Table 1**.

Telehealth usability subscales. ANOVAs revealed non-significant group-differences in the usefulness, ease-of-use, and quality subscales. However, significant group-differences were detected in both the reliability and satisfaction subscales, representing large and moderate effects, respectively. These results are shown in **Table 1**. In this context, reliability represents the extent to which participants felt the intervention supported them consistently and dependably, especially when errors occurred. Satisfaction represents participants’ acceptance, willingness to reuse, and overall positive evaluation of the intervention.

Post-hoc Tukey-Kramer HSD tests showed that for the reliability subscale, both the App-only group (*M* = 5.15, *SD* = 1.01, *p* = .049, *d* = 0.67) and Coach-assisted group (*M* = 5.74, *SD* = 1.08, *p* <.001, *d* = 1.23) reported significantly higher subscale scores than the Newsletter group (*M* = 4.44, *SD* = 1.08). For the satisfaction subscale, the Newsletter group (*M* = 5.75, *SD* = 1.16) reported significantly higher subscale scores than the App-only group (*M* = 4.76, *SD* = 1.23, *p* = .007, *d* = 0.86). No other pairwise comparisons were significant.

### Adherence

Adherence was calculated based on the following metrics: study completion, newsletter opens, diary entries, and adherence to bedtime and wake-up time prescriptions as generated by the CBT-i Coach app. Out of the 79 participants, a total of 8 dropped out of the study (10.13%)—3 from the Newsletter group (3.80%), 4 from the App-only group (5.06%), and only 1 from the Coach-assisted group (1.27%). Overall, 89.87% of participants completed the entire 14-day study. The number of newsletters opened did not differ between groups (Newsletter = 78.88%; App-only = 83.04% and Coach-assisted =73.04%), *F*(2,72) = 0.79, *p* = .458, η^2p^ = .020. The App-only group completed an average of 12.07 (*SD* = 4.18) of the 14 total diary entries, whereas the Coach-assisted group completed an average of 13.48 diaries (*SD* = 2.12). However, there was no statistically significant difference between these groups in total diary entries completed *t*(38.55) = −1.56, *p* = .126, *d* = − 0.42.

Adherence to bedtime and wake-up time prescriptions is summarised in **Table 2**. Participants in the Coach-assisted group were significantly more adherent to both the bedtime and wake-up time prescriptions compared to the App-only group. Specifically, participants receiving coach support deviated from prescribed bed and wake times by approximately half as much as those in the App-only group. Furthermore, those receiving coach assistance were significantly more consistent in applying the bedtime prescription, but even more so, the wake-up time prescription.

**Table 2:** Deviations from the sleep prescription in minutes per group.

Variable	App-only	Coach-assisted	Prescription adherence				Prescription consistency			
	*M (SD)*	*M (SD)*	*t*	*df*	*p*	*d*	*t*	*df*	*p*	*d*
Bedtime	91.29 (58.56)	46.81 (40.66)	2.78	35	.009	0.97	1.72	35	.094	0.60
Wake up time	115.03 (82.71)	48.78 (47.90)	4.29	13	.001	1.91	3.28	35	.002	1.15

*SD*, standard deviation, and represents the consistency with which participants applied the sleep prescription over the intervention period.

### Efficacy

**Table 3** shows intervention and time effects, and their interaction, for all clinical variables.

**Table 3:** Change in symptoms of insomnia, depression and posttraumatic stress in participants completing an App-only, Coach-assisted or newsletter intervention.

Group	Baseline	3 days	7 days	14 days	Time				Group				Time × Group			
	*M (SD)*	*M (SD)*	*M (SD)*	*M (SD)*	*F*	*df*	*p*	η^2p^	*F*	*df*	*p*	η^2p^	*F*	*df*	*p*	η^2p^
Insomnia					162.17	3,234	<.001	.410	0.45	2,76	.640	.012	0.71	2,234	.492	<.001
App-only	14.22 (4.09)	12.48 (4.52)	9.96 (4.06)	9.12 (3.88)												
Coach-assisted	14.07 (3.36)	11.81 (4.26)	8.96 (4.69)	7.26 (5.16)												
Newsletter	14.24 (3.65)	12.68 (4.58)	10.56 (5.08)	7.84 (6.90)												
Depression					130.61	3,234	<.001	.358	2.67	2,76	.076	.066	0.03	2,234	.967	<.001
App-only	16.33 (5.35)	13.11 (4.94)	11.22(5.89)	10.11 (5.98)												
Coach-assisted	13.67 (5.15)	9.81 (4.54)	8.04 (5.38)	7.00 (6.35)												
Newsletter	13.76 (5.52)	11.64 (6.02)	9.48 (7.15)	7.56 (6.43)												
Posttraumatic stress					212.35	2,234	<.001	.476	4.79	2,76	.011	.112	3.80	2,234	.013	.031
App-only	20.78 (4.57)	17.04 (6.65)	14.44 (6.41)	13.04 (7.63)												
Coach-assisted	19.93 (3.75)	15.07 (4.98)	11.37 (5.37)	9.19 (7.07)												
Newsletter	19.72 (4.23)	13.92 (4.65)	10.52 (4.36)	7.24 (4.69)												

Means and standard deviations are based on imputed scores. For all outcomes, N was 78 and the ANOVA statistics were calculated from Mixed Linear Models with a Random intercept for participant. Time was treated as a linear variable with values 0,3,7 and 14 to account for the uneven spacing and linear comparison.

#### Posttraumatic stress

**Figure 1:**
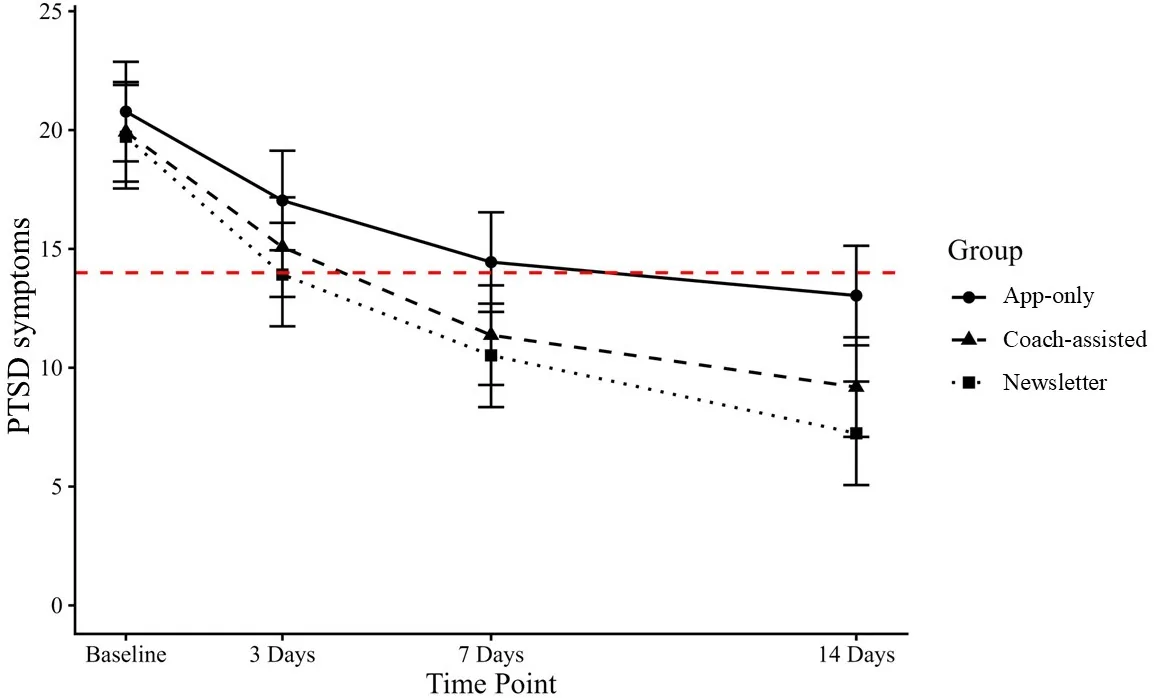
Change in posttraumatic stress symptoms in participants completing an App-only, Coach-assisted or newsletter intervention. Posttraumatic Stress shows mean imputed SPRINT scores, *n* = 78. Reference line at Sprint = 14 showing clinical threshold.

**Figure 1** illustrates posttraumatic stress symptoms in all three groups across the intervention period. A significant, moderate interaction effect was detected, as shown in **Table 3**. Main effects were also significant and are shown in **Table 3**. Post-hoc tests were conducted only for simple main effects owing to the interaction effect.

##### Simple main effects for group

Simple effects analysis comparing groups at each time point showed that there were no significant between-group differences at baseline, 3-days, or 7-days. However, a significant, large simple main effect was detected at 14-days, *F*(2,74) = 6.06, *p* = .015, η^2p^ = .141. A Tukey-Kramer HSD post-hoc test revealed SPRINT scores were significantly higher in the App-only group (*M* = 1.08, *SD* = 0.27) compared both to the group (*M* = 0.86, *SD* = 0.33; *p* = .014, *d* = 0.78) and to the Newsletter group (*M* = 0.84, *SD* = 0.22; *p* = .007, *d* = 0.88).

##### Simple main effects for time

Simple main effects for time. Simple effects analyses comparing changes in each time point for each group showed large simple main effects within each group: App-only, *F*(3,75) = 10.20, *p* <.001, η^2p^ = .290; Coach-assisted, *F*(1.75,43.76) = 28.18, *p* <.001, η^2p^ = .530; and Newsletter, *F*(2.13,49.09) = 51.61, *p* <.001, η^2p^ = .692.

Post-hoc tests revealed that in the App-only group, firstly, SPRINT scores were significantly higher at baseline (*M* = 1.31, *SD* = 0.95) compared both to scores at 7-days (*M* = 1.15, *SD* = 0.18; *p* = .003, *d* = 1.02) and to scores at 14-days (*M* = 1.08, *SD* = 0.27; *p* <.001, *d* = 1.48). Secondly, scores were significantly higher at 3-days (*M* = 1.21, *SD* = 0.20) compared to those at 14-days (*p* = .019, *d* = 0.83).

In the Coach-assisted group, firstly, SPRINT scores were significantly higher at baseline (*M* = 1.29, *SD* = 0.08) compared to all subsequent time points: 3-days (*M* = 1.16, *SD* = 0.15; *p* = .038, *d* = 0.76), 7-days (*M* = 1.05, *SD* = 0.19; *p* <.001, *d* = 1.37), and 14-days (*M* = 0.86, *SD* = 0.34; *p* <.001, *d* = 2.46). Secondly, scores were significantly higher at 3-days (*p* <.001, *d* = 1.70) and 7-days (*p* = .001, *d* = 1.09) compared to those at 14-days.

In the Newsletter group, all pairwise comparisons were significant, such that scores were significantly higher at each time point compared to all subsequent time points: baseline (*M* = 1.28, *SD* = 0.09) compared to 3-days (*M* = 1.14, *SD* = 0.12; *p* = .003, *d* = 1.06), 7-days (*M* = 1.00, *SD* = 0.17; *p* <.001, *d* = 2.20), and 14-days (*M* = 0.84, *SD* = 0.22; *p* <.001, *d* = 3.41); 3-days compared to 7-days (*p* = .001, *d* = 1.14) and 14-days (*p* <.001, *d* = 2.35); and 7-days compared to 14-days (*p* <.001, *d* = 1.21).

In summary, regarding the interaction between group and time, participants in the Coach-assisted and Newsletter groups had a clear decrease in posttraumatic symptoms between time points, which was less pronounced for the App-only group, where there were fewer improvements between each time point. Furthermore, by the end of the intervention period, those in the App-only group had a higher burden of posttraumatic symptoms than those in the other groups.

#### Insomnia

**Figure 2:**
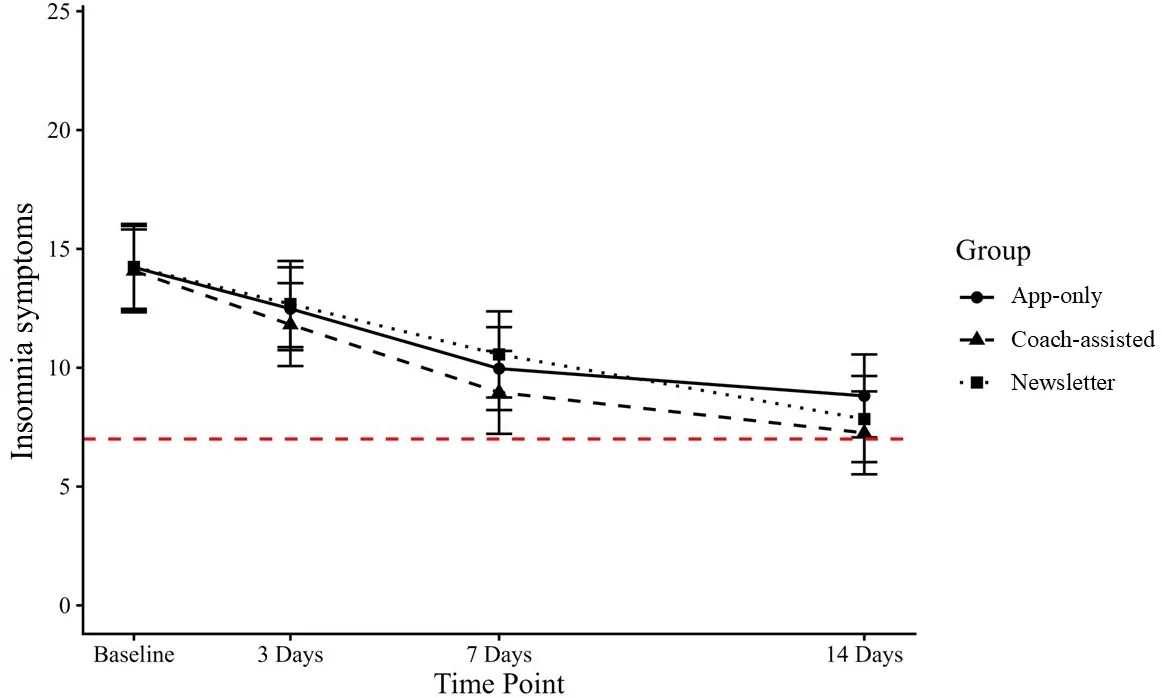
Change in insomnia symptoms in participants completing an App-only, Coach-assisted or newsletter intervention. Insomnia shows mean imputed ISI scores, *n* = 78. Reference line at ISI = 7 representing clinical threshold.

**Figure 2** illustrates insomnia symptoms in all three groups, across the intervention period.

##### Main effect for time

Main effect for time. As indicated in **Table 3**, the between-subjects main effect and interaction effect were both non-significant, but a significant and large within-subjects main effect was detected. A Tukey HSD post-hoc test showed that all comparisons were significant (*p* <.001–.002; *d* = 0.57–1.90). This indicates that, irrespective of group, all participants’ ISI scores were significantly higher at each time point compared to all subsequent time points.

#### Depression

**Figure 3:**
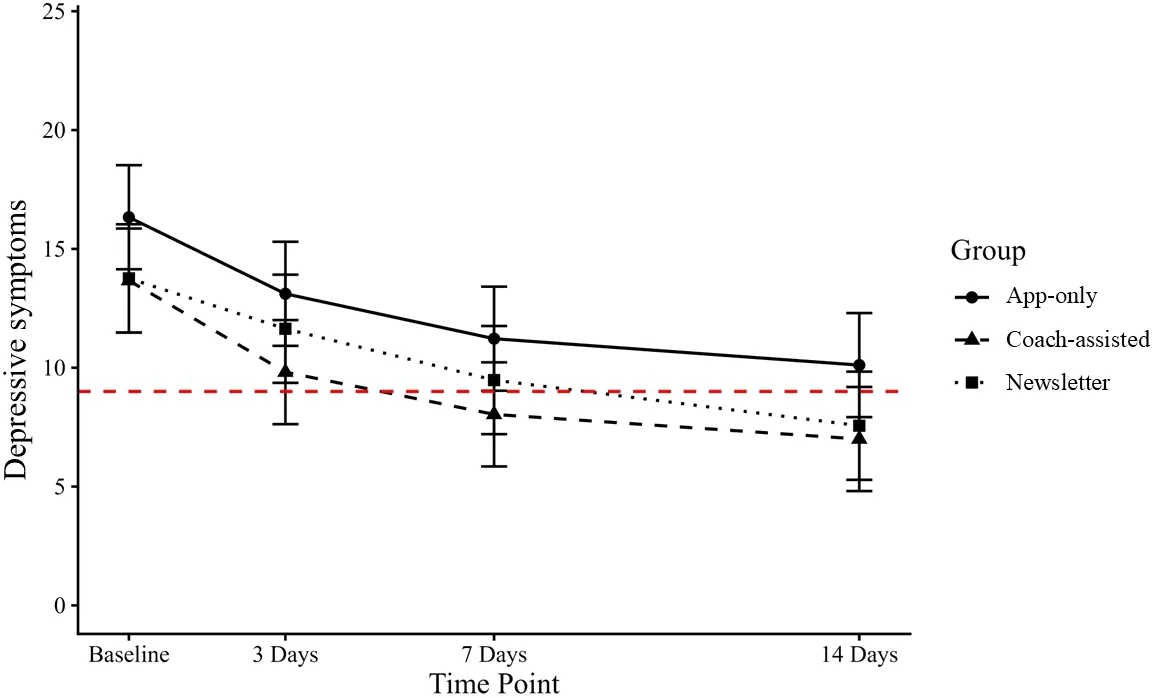
Change in depressive symptoms in participants completing an App-only, Coach-assisted or newsletter intervention. Depression shows mean imputed PHQ-9 scores, *n* = 78. Reference line at PHQ = 9 showing clinical threshold.

**Figure 3** illustrates depressive symptoms in all three groups across the intervention period.

Although there was no significant interaction effect, a significant, moderate main effect was detected between-subjects, although this fell just short of traditional levels of significance. A significant, large main effect was detected within-subjects. These results are shown in **Table 3**.

##### Main effect for group

A Tukey-Kramer HSD post-hoc test revealed that, across all time points, PHQ-9 scores were significantly higher in the App-only group (*M* = 3.49, *SD* = 0.64) compared to the Coach-assisted group (*M* = 2.97, *SD* = 0.79; *p* = .039, *d* = 0.68).

##### Main effect for time

A Tukey HSD post-hoc test revealed that, across all groups, all pairwise comparisons between time points were significant (*p* <.001–.008; *d* = 0.51–1.92), with the exception of the 7- and 14-day comparison (*p* = .055). This indicates that PHQ-9 scores were significantly higher at each time point compared to all subsequent time points, up until the last time point.

## Discussion

We evaluated a coach-assisted dHealth application in comparison with a self-guided version of the application, in the context of standard stimulus control and sleep hygiene information administered to all participants. Outcomes included usability, the presence of adverse events, and adherence to each intervention, as well as preliminary data describing changes in insomnia, depression, and PTSD-related symptoms during the intervention period.

Regarding participant experiences of the interventions, of the two application-based therapies, women using the application without support reported more intervention-related negative effects and less satisfaction than those receiving the newsletter only.

Regarding adherence, there were no differences with regards to treatment non-completion, newsletter engagement or application-specific diary entries. However, participants receiving coach support, in comparison to those using the application without guidance, were more adherent to the sleep prescription regarding deviations from it, and in consistency in applying it.

Regarding symptom profiles, our pilot results showed that PTSD symptoms decreased more prominently for participants using the application together with coach assistance and those receiving the daily newsletter only. Those who used the application without assistance showed an initial response to the intervention, with subsequent symptom improvement reaching a plateau. Participants in this group reported PTSD symptoms at the threshold level at the end of the trial, while participants in the other groups showing substantially fewer symptoms that were below threshold at 14 days. Insomnia symptoms decreased irrespective of the treatment. Regarding depressive symptoms, while all participants showed an improvement in their symptoms over the intervention period, participants using the application without support had overall higher levels of depression over the study period, despite not reporting higher levels of depression at baseline.

### Application-specific negative effects and usability

Our findings showed that, in terms of intervention-specific negative effects, participants using the dHealth application without support reported more negative effects than those receiving only the newsletter. The difficulty of implementing a substantial behavioural change self-guided - reducing sleep opportunity while experiencing ongoing insomnia symptoms - may have contributed to these effects. Previous research has shown that sleep rescheduling is associated with mood changes, fatigue, sleepiness, and irritability (73) and can be challenging to implement even in in-person clinician-patient settings (30).

There were no differences in negative effects between participants receiving coach-assisted dHealth support and those receiving only the newsletter. These findings suggest that both interventions were acceptable to participants and indicate that a trained sleep coach may help mitigate the negative effects associated with sleep rescheduling.

Regarding usability, the findings showed that participants using the application with or without support reported better reliability than participants receiving the newsletter only. This is likely because reliability described whether the technology had reliable systems in place for application-related errors, which are not relevant to newsletter engagement.

More pertinently, participants using the application without coach assistance reported lower satisfaction than participants reading the newsletter. One reason for this result may be related to the fact that implementing the intervention is unpleasant: curtailing sleep opportunity while experiencing insomnia and psychiatric symptoms is difficult (74). In contrast, reading a newsletter and implementing stimulus control and sleep hygiene information may be more accessible when the intervention is self-administered. Participants using the application with support did not differ in their satisfaction ratings from those receiving the newsletter, suggesting that using coach assistance rescued the decrease in satisfaction associated with using the application independently.

### Adherence to the dHealth application

Adherence was assessed both broadly and specifically. General measures included dropout rates, newsletter engagement, and application-based sleep diary completion, while targeted measures evaluated adherence to the prescribed sleep schedule, the core therapeutic component of the intervention. Each intervention type had similar rates of drop-out, newsletter engagement and application-specific sleep diary entries. Notably, adherence rates regarding study completion, newsletter engagement, and use of the application were significantly higher than those typically reported for individuals using self-administered dHealth interventions (33), likely because participants were enrolled in a study that directly and indirectly encouraged completion.

However, there were notable differences between those using the application with and without coach support in adherence to the sleep prescription, the active ingredient in sleep rescheduling. Notably, deviations from the prescription were large in both groups. However, those receiving coach assistance deviated from the prescribed bedtime by half of that indicated by self-guided participants. This result was similar and more pronounced for the prescribed wake-up time, indicating that without coach support, waking up at a preferred time is very difficult when completing a sleep rescheduling regimen – participants tended to wake up almost 2 hours outside of their prescribed bedtime, in comparison to approximately a 50-minute deviation when supported by a coach. The same pattern of results was observed for the consistency with which participants implemented the prescription: those receiving application-related coach assistance were more consistent in applying the prescription for both bedtime and wake-up time, with larger effects for the latter time. These results highlight that coach assistance is critical for the implementation of sleep rescheduling. Without adherence to the sleep prescription, individuals cannot reap the benefits of this proven component of CBT-i treatment.

### Coach-assisted augmentation of a sleep-focused dHealth application for symptoms of insomnia, depression and PTSD

All efficacy findings are considered preliminary, because of the small sample size and short intervention period. Regarding posttraumatic symptoms, our findings showed that participants receiving coach assistance while using the application, and those receiving the newsletter only, had a consistent reduction in their symptoms over the two-week period. By the end of the intervention period, participants using the application without assistance had significantly higher posttraumatic symptoms than individuals in the other groups. While the dHealth intervention focused on delivering sleep therapy, leveraging sleep rescheduling, the newsletter informed participants about improving their bed and sleep association (stimulus control) and altering their sleep environment to optimise their sleep (sleep hygiene).

Regarding insomnia symptoms, participants in this study reported, on average, subclinical levels, although nearly half the sample met the threshold for probable clinical insomnia at baseline. Across conditions, insomnia symptoms decreased by the end of the two-week intervention. Participants receiving coach-assisted support and those in the newsletter-only condition, on average, showed minimal or no residual insomnia symptoms. In contrast, participants using the application without assistance showed reduced but still subthreshold symptoms.

These findings are broadly consistent with prior research. For example, a recent study evaluating a dHealth application based on CBT-i principles over an 18-day period found that 132 adults with insomnia experienced reductions in insomnia, depression, and anxiety symptoms that were maintained at three-week follow-up (75). Compared with a waitlist control group, participants in the intervention arm showed improved sleep efficiency based on both self-report and actigraphy. Notably, that intervention did not include sleep rescheduling, but instead incorporated sleep hygiene, stimulus control, wind-down techniques, and mindfulness.

Together, our findings and those of Staiano and colleagues, suggest that insomnia symptoms can be reduced to non-clinical levels using interventions that include stimulus control and sleep hygiene—components that all participants in our study received. This is further supported by meta-analytic evidence showing that digital CBT-i significantly reduces insomnia symptoms, including among individuals exposed to trauma (76).

Regarding depressive symptoms, all participants showed an improvement in their symptoms over the intervention period. However, participants using the application without coach support had overall higher levels of depression over the study period, despite not reporting higher levels of depression at baseline.

All participants received the newsletter, and the results suggest that combined self-guided stimulus control and sleep hygiene are sufficient to reduce posttraumatic symptoms. This intervention may be effective in a self-guided format because it is relatively easy to implement and places less burden on individuals than sleep rescheduling, which is a more demanding intervention. Furthermore, using a sleep prescription to apply principles of sleep rescheduling, without coach assistance, is associated with a plateau in symptom reduction. Put differently, the efficacy of stimulus control/sleep hygiene is reduced when it is paired with sleep rescheduling without support. The findings suggest that sleep rescheduling should be implemented with coach assistance.

A recent network meta-analysis set out to evaluate which components of CBT-i are associated with improved outcomes, by comparing psychoeducation, sleep rescheduling, stimulus control, cognitive therapy, and relaxation therapy as components of CBT-i (77, 78). The authors found, congruent with an earlier study evaluating sleep rescheduling as a stand-alone therapy (78), that sleep rescheduling was the only component that was associated with a reduction in insomnia symptoms. The authors also found that both sleep rescheduling and stimulus control improved sleep quality and quantity: sleep rescheduling was related to improved self-reported sleep continuity and sleep quality, while stimulus control was associated with improved self-reported and objective total sleep time. However, the meta-analysis did not discuss to what extent the interventions were delivered digitally or in-person or whether sleep rescheduling, or other components of CBT-i, were implemented with or without guidance from a clinician or coach. While these findings highlight the efficacy of sleep rescheduling, our preliminary findings suggest that sleep rescheduling is likely only useful when guided by a coach and that when this treatment pathway is not available, self-guided stimulus control and sleep hygiene can be effective.

Although our study found intervention-specific differences in posttraumatic symptoms, but not in insomnia or depression, similar patterns have been reported in other studies of sleep-focused interventions for individuals with PTSD symptoms. For example, a recent study evaluated a custom-built sleep-focused dHealth application for trauma survivors, delivered as a four-week, six-module programme incorporating cognitive restructuring, sleep hygiene, stimulus control, sleep rescheduling, nightmare rescripting, and mindfulness (79). Thirty participants with posttraumatic symptoms were randomised to either the intervention or a waitlist control group, with subsequent crossover. The programme was self-paced. Results showed significant reductions in posttraumatic and depressive symptoms in the intervention group, but no significant changes in insomnia symptoms.

Furthermore, our sample is located in a LMIC context, and these findings show, that across a range of socio-economic levels, both coach-assisted sleep rescheduling and self-guided stimulus control and sleep hygiene, are potentially effective treatments for symptoms of insomnia, depression and PTSD.

### Limitations and future directions

Our findings are tempered by a number of significant limitations. Firstly, our study is a pilot, with a small sample size, and findings from this study should be confirmed by a larger, well-powered study. In this regard, our small sample had limited power to evaluate interaction effects, especially for secondary outcomes. Our study also evaluated the interventions in a shorter time period than standard CBT-i, and we were not able to conduct long-term follow-up. While our results indicate that coach-assisted sleep rescheduling and self-guided stimulus control and sleep hygiene are equally effective treatments, our study was not able to show whether the symptom improvements were maintained over time. A recent study examining the long-term efficacy of CBT-i delivered either in person or via a self-guided dHealth application found that, at both 9 weeks (post-intervention) and 33 weeks (long-term follow-up), sleep-related outcomes were worse in the digital application group than in those receiving in-person treatment (80). Future research should therefore assess intervention outcomes, usability, and adherence over longer periods, including follow-up after treatment completion, particularly in dHealth applications that include coach support.

Secondly, our study was not designed to test interactions between guidance (coach-assisted vs self-guided delivery) and all CBT-i components. As a result, we were unable to fully evaluate these effects. Instead, our findings highlight the need for future research to consider not only coach augmentation in general, but also which specific components of CBT-i may require coaching to achieve optimal outcomes.

Thirdly, we note that the reliability of the ISI and SPRINT were low at baseline. The initial low reliability may have occurred because the measures were included in the screening pack, before participants engaged more actively with the research materials (reliability post-baseline was acceptable for all outcomes). We also note that for the groups obtaining the dHealth intervention, the active ingredient of the sleep prescription was delivered on day 5 of the intervention. Participant symptom evaluation on day 3 was done prior to the delivery of the intervention active ingredient, suggesting that the significant comparisons between day 3 and other time points represent a pre- to during/post-intervention comparison, lending credibility to interpretations of symptom improvement for those comparisons. Nonetheless, we highlight that results related to changes in PTSD and insomnia symptoms, should be interpreted with caution.

Fourthly, the generalisability of our findings is limited by our target sample of young, female, tertiary-level students. These findings may not generalise to other populations, although we do note that this LMIC-based sample represents an under-studied and under-represented population. Regarding studies that have been conducted in men, there is a paucity of data on male responses to dHealth interventions, especially in LMICs (81). and replication with male participants is important to inform targeted treatment. Regarding older adults, aggregate meta-analytic findings show that dHealth interventions can be effective in older age for anxiety-related conditions (82), but there are also significant barriers to use, as older adults may not be familiar with, or desire to access treatment, using technological resources (83).

Lastly, our study, and future studies evaluating sleep interventions, would benefit from measuring objective sleep measurement through actigraphy or polysomnography, to compliment self-reported changes in sleep duration and quality.

## Conclusion

Together, these results indicate that participants applying sleep rescheduling without coach support report less favourable usability ratings, more intervention-related negative effects, and have poorer adherence to the sleep prescription. Preliminary efficacy results suggest potential poorer outcomes, including higher PTSD symptoms at the end of the intervention, compared with those receiving coach-assisted support or simple stimulus control and sleep hygiene information.

These results suggest that sleep rescheduling may be difficult to implement effectively without coach support. However, when supported sleep rescheduling is unavailable, self-guided stimulus control and sleep hygiene appear to be acceptable interventions, with high satisfaction, few negative effects, and potential benefits for reducing PTSD symptoms.

Future studies should directly compare all CBT-i components delivered digitally over longer follow-up periods to replicate and extend these findings.

## Data Availability

The raw data supporting the conclusions of this article will be made available by the authors, without undue reservation.
